# CARDIOMETABOLIC DISEASE RISK PARTIALLY MEDIATES RELATIONSHIP BETWEEN FINE PARTICULATE MATTER AND COGNITION

**DOI:** 10.1093/geroni/igae098.3038

**Published:** 2024-12-31

**Authors:** Ethan Siu Leung Cheung, Sara Grineski, David Curtis

**Affiliations:** University of Utah, Salt Lake City, Utah, United States; University of Utah, Salt Lake City, Utah, United States; University of Utah, Salt Lake City, Utah, United States

## Abstract

PM2.5 pollution is expected to worsen in many places due to climate change. This is because of hotter temperatures, less precipitation, and increases in windspeed. PM2.5 exposure is associated with many adverse health effects. Less is known about how physical and mental health challenges associated with aging, like the onset of cardiometabolic disease (CMB) and changes in psychological wellbeing, might mediate linkages between PM2.5 exposure and aging-related cognitive and physical limitations. Longitudinal data from the Midlife in the United States study (MIDUS 1: 2004-05; MIDUS 2: 2013-14) were adopted. A sample of 4,272 individuals aged 32 to 84 was included. Based on individuals’ residential addresses at each wave, we linked census tract identifiers to PM2.5 exposure, which was defined by five-year annual averages. Using structural equation models, we examined cross-sectional and longitudinal associations between PM2.5 exposure and executive functioning and instrumental activities of daily living (IADL) limitations, and tested CMB risk and psychological well-being as mediators using the product of coefficients method. Results suggested that higher PM2.5 exposure was associated with lower executive functioning cross-sectionally and with larger decreases over time. No significant associations were found between PM2.5 and IADL limitations. The cross-sectional association between PM2.5and executive functioning was partially mediated by CMB risk, accounting for 14% of the estimate, and the longitudinal association showed a similar, albeit nonsignificant mediation pattern. Psychological wellbeing was not a significant mediator. Findings suggest the importance of considering the complex and indirect ways in which climate change may impact health of older adults.

